# Preparation of Alginate Hydrogel Beads on a Superhydrophobic Surface with Calcium Salt Powder to Enhance the Mechanical Strength and Encapsulation Efficiency of Ingredients

**DOI:** 10.3390/ma17246027

**Published:** 2024-12-09

**Authors:** Yuhei Hosokawa, Takashi Goshima, Takami Kai, Saki Kobaru, Yoshihiro Ohzuno, Susumu Nii, Shiro Kiyoyama, Masahiro Yoshida, Takayuki Takei

**Affiliations:** 1 Department of Chemical Engineering, Graduate School of Science and Engineering, Kagoshima University, 1-21-40 Korimoto, Kagoshima 890-0065, Japan; k7857533@kadai.jp (Y.H.); tgoshima@cen.kagoshima-u.ac.jp (T.G.); takkai@cb4.so-net.ne.jp (T.K.); inamine@eng.kagoshima-u.ac.jp (S.K.); ohzuno@cen.kagoshima-u.ac.jp (Y.O.); niisus@cen.kagoshima-u.ac.jp (S.N.); myoshida@cen.kagoshima-u.ac.jp (M.Y.); 2 Department of Chemical Science and Engineering, National Institute of Technology, Miyakonojo College, 473-1 Yoshio, Miyazaki 885-8567, Japan; shiroh@miyakonojo-nct.ac.jp

**Keywords:** hydrogel, bead, capsule, alginate, drug, water-repellent surface

## Abstract

Calcium alginate hydrogel is one of the most widely used materials for drug-carrier beads used in drug-delivery systems. In this study, we developed a new method to improve the encapsulation efficiency of ingredients, such as medicines, in calcium alginate hydrogel beads. In the gold standard method, the hydrogel beads are prepared in the liquid phase. In contrast, in the new method, to enhance the encapsulation efficiency, the hydrogel beads are prepared in the gas phase using a water-repellent surface. In brief, a droplet of sodium alginate aqueous solution is rolled on a water-repellent surface with CaCl_2_ powder, a cross-linking agent. This process leads to the direct attachment of CaCl_2_ powder to the droplet, resulting in the formation of spherical hydrogel beads with high mechanical strength and higher encapsulation efficiency than beads prepared by previous methods. The hydrogel beads exhibit similar permeability for glucose, a model for low-molecular-weight medicines, to those prepared by previous methods. These results show that the new method is promising for the preparation of calcium alginate hydrogel beads for drug-delivery systems.

## 1. Introduction

Hydrogels are used for various applications in the medical, food, and environmental fields because of their high water content and biocompatibility [1,2,3,4,5], and hydrogel beads are used as drug carriers in drug-delivery systems (DDSs) [6,7,8,9]. Calcium alginate hydrogel is one of the most widely used materials for the drug-carrier beads in DDSs [10,11]. This is because calcium alginate hydrogel is biocompatible and can be prepared under mild physiological conditions [12,13,14].

The gold standard method for preparing calcium alginate hydrogel beads is the liquid curing method, in which an aqueous solution of sodium alginate containing ingredients, such as medicines, is dropped into an aqueous solution (curing liquid) containing Ca^2+^ as a cross-linking agent for the alginate polymer [15,16,17,18,19,20,21,22]. This results in the rapid formation of a stable cross-linking structure, which is often described as an “egg-box model”, resulting in the rapid formation of calcium alginate hydrogel beads immediately after the alginate solution droplets leach into the curing liquid [23]. In this paper, this method is referred to as PM1. Hydrogel beads can be prepared by PM1 in a single and simple step. However, this method often causes leakage of the ingredients from the droplets into the curing liquid. Therefore, it is difficult to efficiently encapsulate ingredients in hydrogel beads prepared by this method. This is because the gelation is achieved in liquid, which has a high ability to dissolve substances owing to its high molecular density. If the gelation could be achieved in the gas phase, in which the molecular density is much lower than that in the liquid phase, it is expected that highly efficient encapsulation of ingredients could be achieved. Mano and co-workers [24] and our group [25] have demonstrated that it is possible to achieve ultra-efficient encapsulation of ingredients in millimeter-sized capsules or beads in the gas phase by solidifying spherical liquid droplets on a lotus-leaf-like superamphiphobic surface. Subsequently, we have fabricated capsules and beads in the gas phase using various polymers and capsule solidification methods [26]. Furthermore, we have demonstrated that the combination of interfacial thermodynamic theory (equilibrium theory) and the centrifugal force (kinetics) allows for precise control over the core–shell structure of the capsule [27]. There have been a few reports on the preparation of calcium alginate hydrogel beads in the gas phase using a superamphiphobic surface setup. Mano and co-workers [24,28] prepared hydrogel beads by adding a small amount of an aqueous solution containing Ca^2+^ dropwise to spherical sodium alginate solution droplets formed on a superhydrophobic surface. In this paper, this method is referred to as PM2. The potential problem with preparing calcium alginate hydrogel beads by PM2 is that it is difficult to achieve high encapsulation efficiency of ingredients for the same reason as PM1, that is, because a curing liquid is used for the gelation of the alginate solution droplets. Song reported a method to overcome the issue [29]. In their method, spherical droplets are prepared by adding an aqueous solution containing low concentrations of both sodium alginate and Ca^2+^ dropwise to a water-repellent surface, and the water in the droplets is then evaporated to increase the polymer and Ca^2+^ concentrations in the droplet. They found that increasing the concentrations of the polymer and Ca^2+^ resulted in gelation of the droplets, thereby producing calcium alginate hydrogel beads, and the beads showed a high encapsulation efficiency of 88% [29]. In this paper, this method is referred to as PM3. However, it is important to further improve the encapsulation efficiency. Considering that the initial concentrations of alginate and Ca^2+^ are low in PM3, this method produces soft hydrogel beads. The soft hydrogel beads tend to adhere to the water-repellent surface, causing parts of the beads’ surfaces to peel off, which makes it difficult to achieve an encapsulation efficiency of above 90%.

**Figure 1:**
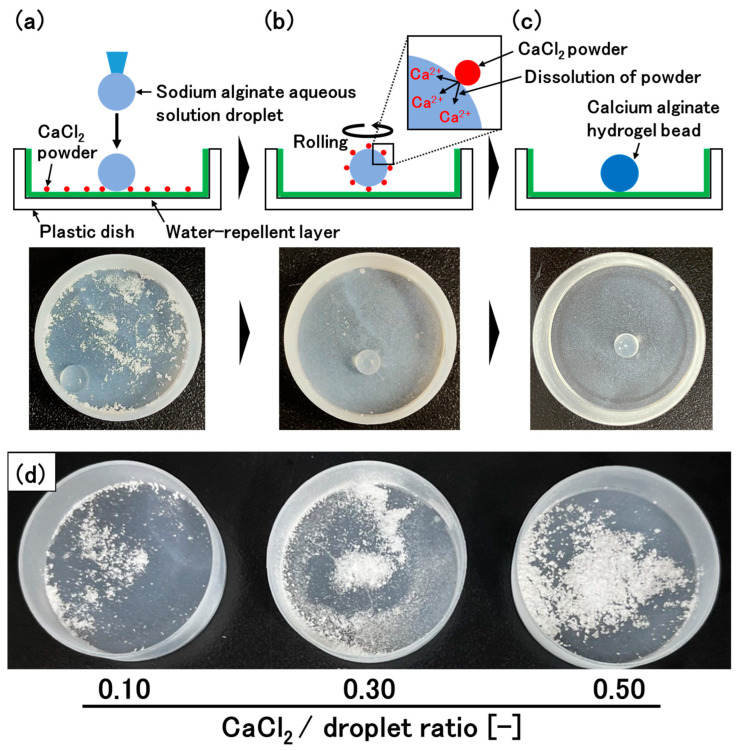
Concept for preparing calcium alginate hydrogel beads with high mechanical strength. (**a**) Spherical droplets are produced by adding an aqueous solution of sodium alginate dropwise onto a water-repellent surface with CaCl_2_ powder. (**b**) By rolling the droplet on the surface, the CaCl_2_ powder attaches to the surface of the droplet. (**c**) The CaCl_2_ powder then dissolves, resulting in gelation of the droplet. (**d**) Amount of CaCl_2_ powder in each CaCl_2_/droplet ratio.

In this study, we aimed to increase the encapsulation efficiency of ingredients in hydrogel beads by improving PM3. To achieve this, it is necessary to fabricate hydrogel beads with high mechanical strength, as described above. In the new method, to achieve high mechanical strength, spherical droplets are first produced by adding an aqueous solution of sodium alginate dropwise onto a water-repellent surface (Figure 1a). The most significant feature of the new method is that powder of a calcium salt is also placed on the surface. By rolling the droplet on the surface, the powder of the calcium salt attaches to the surface of the droplets and then dissolves in the water at the surface, resulting in the generation of Ca^2+^ and the subsequent gelation of the surface of the droplet (Figure 1b,c). The ions diffuse inside the beads, which leads to the gelation of not only the surface, but also the inside of the beads. Owing to the high molecular density of the solid-state cross-linking agent, this method can create a strongly cross-linked structure, leading to the production of calcium alginate hydrogel beads with high mechanical strength, which contributes to an increase in the encapsulation efficiency. Additionally, the diameter of the hydrogel beads can be easily controlled because this method does not require water evaporation. We optimized the experimental conditions to produce spherical hydrogel beads and evaluated various physiological properties of the hydrogel beads.

## 2. Materials and Methods

### 2.1. Materials

Sodium alginate was purchased from Kimica Corporation (Tokyo, Japan). CaCl_2_, glucose, and Glucose CII-Test Wako were purchased from Wako Pure Chemical Industries (Osaka, Japan). Ultra Ever Dry (UED) was purchased from UltraTech International Inc. (Jacksonville, FL, USA). A water-repellent surface was prepared by coating the bottom and side wall of a plastic Petri dish (bottom diameter of 35.6 mm) with UED according to the procedure described in the UED instructions.

### 2.2. Preparation of the Calcium Alginate Hydrogel Beads

The hydrogel beads were prepared by the following procedure. An aqueous sodium alginate solution (3% (*w*/*v*)) was prepared by dissolving sodium alginate in distilled water. CaCl_2_ powder (particle size of 23 ± 22 µm) was evenly spread on the bottom of the UED-coated Petri dish. A droplet of the alginate solution was added to the Petri dish, and the Petri dish was shaken with rotation (MMS-3020, Tokyo Rikakikai Co., Ltd., Tokyo, Japan). The amount of CaCl_2_ powder in the Petri dish is defined as the CaCl_2_/droplet ratio (weight (grams) of CaCl_2_ powder/weight (grams) of the droplet of alginate solution)) (Figure 1d). The size of the droplet, rotation rate of the Petri dish, and rotation time (gelation time) were changed in each experiment. For PM1, the hydrogel beads were prepared using the following procedure [15]. A droplet of 3% (*w*/*v*) sodium alginate solution was added to a CaCl_2_ aqueous solution, and the solution was stirred for gelation. The size of the droplet, volume of the CaCl_2_ aqueous solution, CaCl_2_ concentration, and gelation time were changed in each experiment. For PM2, the hydrogel beads were prepared according to the report by Mono and co-workers [28]. A spherical droplet (0.1 g) of 3% (*w*/*v*) alginate solution was formed on a UED-coated Petri dish and then the same volume of CaCl_2_ aqueous solution as that of the alginate solution droplet was added to the alginate solution droplet. The construct was allowed to rest for gelation. The appearance of the prepared beads was observed with a digital microscope (ADX-AD208-JP, ShenZhen Andonstar Technology Co., Ltd., Shenzhen, China). The sphericity of the hydrogel beads was defined as follows:

Bead sphericity (%) = (shortest diameter of the bead/longest diameter of the bead) × 100

### 2.3. Optimization of the Rotation Rate of the Petri Dish to Prepare Spherical Hydrogel Beads

For optimization of the rotation rate of the Petri dish, the weight of the droplet of alginate solution, CaCl_2_/droplet ratio, and gelation time were fixed at 0.1 g, 0.1, and 1 h, respectively. The rotation rate of the Petri dish (125–250 rpm) was changed to prepare spherical hydrogel beads.

### 2.4. Determination of Gelation Time Required for Sufficient Gelation

For the new method, the weight of the droplet of alginate solution, CaCl_2_/droplet ratio, and rotation rate were fixed at 0.1 g, 0.1, and 200 rpm, respectively. For PM2, the hydrogel beads were prepared by adding 20 droplets (0.1 g each) of alginate solution to 1 L of 0.1 M CaCl_2_ solution. The repulsion force of the hydrogel beads was measured at 30% strain using a tabletop material tester (compression rate of 10 mm/min, MCT-2150, A&D Co., Ltd., Tokyo, Japan). The relationship between the gelation time and the repulsion force of the beads was determined for both methods.

### 2.5. Measurement of Mechanical Strength

The mechanical strengths of calcium alginate hydrogel beads prepared by the new method and by PM1 were measured. For both methods, the gelation time of the droplets of the alginate solution was fixed at 1 h. Because the strength of hydrogel beads depends on their diameter, beads with almost the same diameters were prepared for each set of experimental conditions by changing the droplet size of the alginate solution, CaCl_2_/droplet ratio, and CaCl_2_ concentration of the CaCl_2_ aqueous solution (Table 1). The repulsion force of the hydrogel beads at 50% strain was measured using the same tabletop material tester used to determine the gelation time for sufficient gelation (compression rate of 1 mm/min).

### 2.6. Determination of the Encapsulation Efficiency of Glucose

To investigate the encapsulation efficiency of glucose in the hydrogel beads, glucose was dissolved in alginate solution at a concentration of 40% (*w*/*v*). The polymer solution was used to prepare glucose-encapsulated hydrogel beads. For the new method, the weight of the droplet of alginate solution, CaCl_2_/droplet ratio, and rotation rate were 0.1 g, 0.1–0.5, and 200 rpm, respectively. After the hydrogel beads were removed from the Petri dish, 3 mL of 5 mM 4-(2-hydroxyethyl)-1-piperazineethanesulfonic acid (HEPES) buffer solution (pH 7.0) was added to the Petri dish, and the Petri dish was shaken for 1 h to dissolve the glucose remaining in the Petri dish. The glucose concentration in the aqueous solution was measured by the enzymatic colorimetric method using a glucose measurement kit (Glucose CII-Test Wako). For PM1, a droplet (0.1 g) of glucose-containing alginate solution was added to 0.1 M CaCl_2_ solution (10 mL, pH 7.0) containing 5 mM HEPES, and the solution was stirred for 20 min for sufficient gelation. The glucose concentration in the CaCl_2_ solution was then measured. For PM2, after the hydrogel beads were prepared, the beads were removed from the Petri dish, and the remaining CaCl_2_ solution was completely dried. Next, 3 mL of 5 mM HEPES buffer solution (pH 7.0) was added to the Petri dish, and the Petri dish was shaken for 1 h to dissolve the glucose remaining in the Petri dish in the buffer solution (the same procedure as that for the new method). The absorbance of the sample treated with the glucose measurement kit is dependent on the pH of the sample solution. Therefore, HEPES was used to maintain the pH of the sample solution at pH 7.0.

### 2.7. Permeability Evaluation

The permeability of the beads to glucose was evaluated for calcium alginate hydrogel beads prepared by the new method and by PM1. The weight of the droplet of alginate solution, CaCl_2_/droplet ratio, rotation rate, and gelation time were fixed at 0.1 g, 0.1, 200 rpm, and 1 h, respectively. For PM1, the hydrogel beads were prepared according to the procedure described in Section 2.4. The permeability of the beads to glucose was evaluated by adding the hydrogel beads (2.5 mL, 2.3 mm in diameter for the new method and 2.6 mm in diameter for PM1) to a well-stirred 5 mM HEPES buffer solution (5 mL, 20 °C, pH 7.0) containing 5.3 mg/mL glucose. Aliquots (10 µL) of the solution were collected at various times to measure the concentration of glucose. Under the assumption that the volume of the solution was finite, the concentration of glucose in the solution was uniform, and the structure inside the gel beads was homogeneous, the effective diffusion coefficient of glucose ( $D_{e}$ ) was determined by the nonlinear least-squares method using the following equation [30]:

$$C_{L}=\frac{\alpha C_{L0}}{1+\alpha }\left\{1+\sum_{n=1}^{\infty }\frac{6(1+\alpha )\exp(-D_{e}q_{n}^{2}t/a^{2})}{9+9\alpha +q_{n}^{2}\alpha^{2}}\right\}$$

where $\alpha =(V/n)/(4\pi a^{3}K/3)$ ; $q_{n}$ is defined as $\tan\left(q_{n}\right)=3q_{n}/(3+\alpha q_{n}^{2})$ ; a is the diameter of the hydrogel bead; t is the time; $C_{L0}$ is the initial concentration of glucose; $C_{L}$ is the concentration of glucose in the solution at time *t*; V is the volume of the solution; and n is the number of hydrogel beads. K is the partition coefficient, which is defined as the ratio of the equilibrium concentration of glucose in the hydrogel beads to that in the solution.

## 3. Results and Discussion

The most significant feature of the new method to prepare calcium alginate hydrogel beads is that powder of a calcium salt, a cross-linking agent for the alginate polymer, is placed on a water-repellent surface (Figure 1a). By rolling the droplet on the surface, the powder of the calcium salt attaches to the surface of the droplets and then dissolves in the water at the surface, resulting in the generation of Ca^2+^ and the subsequent gelation of the surface of the droplet (Figure 1b,c). The ions diffuse inside the beads, which leads to gelation of not only the surface, but also the inside of the beads. Because of the high molecular density of the solid-state cross-linking agent, it is expected that the new method can produce strongly cross-linked calcium alginate hydrogel beads with high mechanical strength, resulting in high encapsulation efficiency. In this study, the alginate concentration of the polymer solution was fixed at 3% (*w*/*v*) for the new method, as well as for PM1 and PM2.

### 3.1. Optimization of the Rotation Rate of the Petri Dish to Prepare Spherical Hydrogel Beads

**Figure 2:**
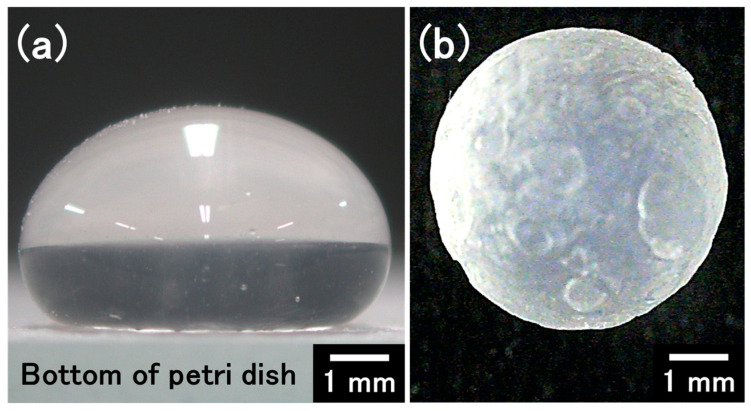
(**a**) A droplet (0.1 g) of sodium alginate aqueous solution on the water-repellent surface. (**b**) A calcium alginate hydrogel bead prepared by the new method (droplet weight of 0.1 g, rotation rate of the Petri dish of 200 rpm, and rolling time of 1 h).

**Figure 3:**
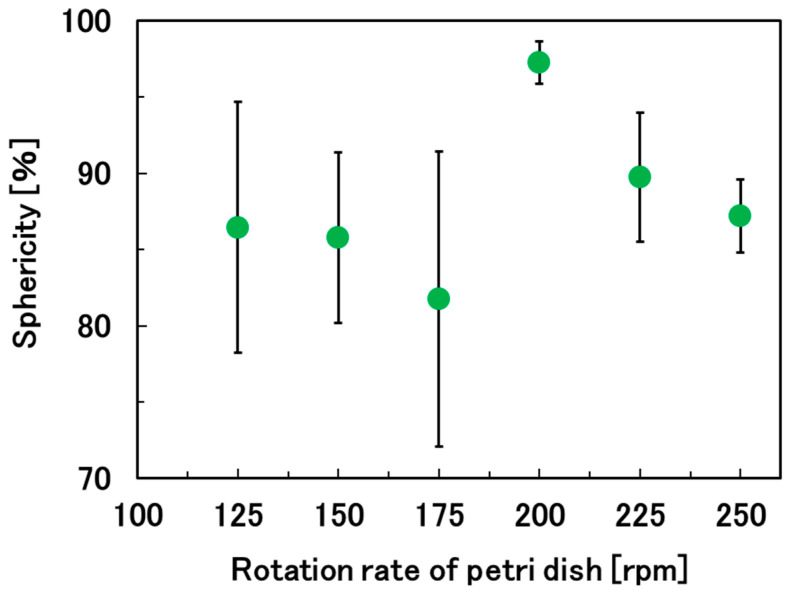
Relationship between the rotation rate of the Petri dish and the sphericity of the calcium alginate hydrogel beads.

By using UED, we were able to create a suitable water-repellent surface to prepare a droplet that could freely roll on the surface (Figure 2a). As expected, the powder of the calcium salt attached to the surface of the droplets and then dissolved in the water at the surface, resulting in gelation of the droplet (Figure 1a–c and Figure 2b). Next, we optimized the rotation rate of the Petri dish (125–250 rpm) to produce spherical calcium alginate hydrogel beads. In this experiment, the weight of the droplet of alginate solution, the CaCl_2_/droplet ratio, and the rolling time of the droplet (gelation time) were fixed at 0.1 g, 0.1 and 1 h, respectively. At a rotation rate of 0 rpm, the gravitational force overcame the interfacial tension, leading to distorted droplets (Figure 2a). However, at a rotation rate of 200 rpm, the appropriate centrifugal force pushed the droplets against the side of the Petri dish, resulting in the formation of spherical hydrogel beads (Figure 2b and Figure 3). At a lower rotation rate (125 rpm), the sphericity of the beads decreased owing to the irregular rolling of the droplets. At a rotation rate higher than 200 rpm, excess centrifugal force was applied to the droplets, resulting in rugby-ball-like beads. In the subsequent experiments, the rotation rate was fixed at 200 rpm.

### 3.2. Optimization of the Gelation Time for Sufficient Gelation of the Hydrogel Beads

**Figure 4:**
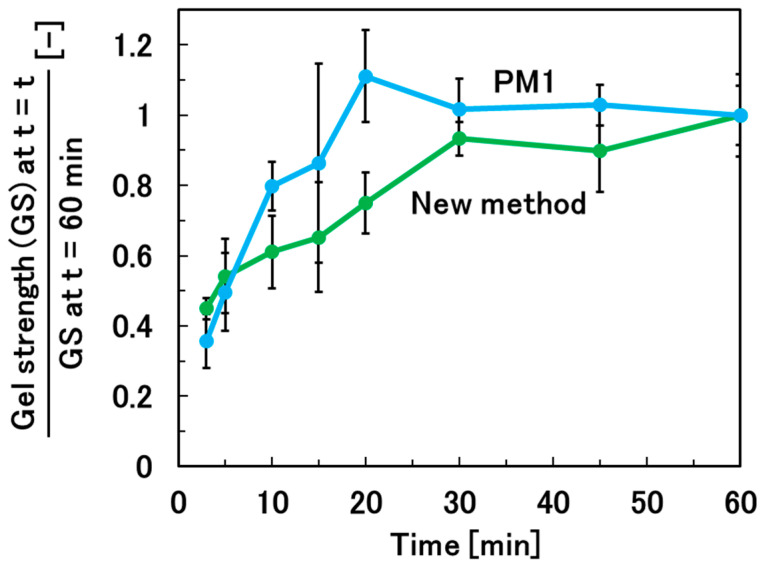
Relationship between the gelation time and mechanical strength of calcium alginate hydrogel beads prepared by the new method and by PM1 to determine the time required for sufficient gelation.

The gelation time required for sufficient gelation of the alginate solution droplets was investigated. The new method was compared with PM1, the gold standard method for the preparation of hydrogel beads. For the new method, the weight of the droplet of alginate solution, the CaCl_2_/droplet ratio, and the rotation rate were fixed at 0.1 g, 0.1, and 200 rpm, respectively. For PM1, the hydrogel beads were prepared by adding 20 droplets (0.1 g for each) of alginate solution dropwise to 1 L of CaCl_2_ solution. Sufficient gelation was achieved in over 30 min for the new method and in over 20 min for PM1 (Figure 4). The reason why the new method takes longer to achieve sufficient gelation than PM1 is that the new method requires the dissolution of the CaCl_2_ powder at the droplet surface. It is expected that sufficient gelation can be achieved with a gelation time of over 20 min for PM2, which uses CaCl_2_ solution for the gelation of the alginate solution droplets, similar to PM1.

### 3.3. Mechanical Strength of the Hydrogel Beads

**Figure 5:**
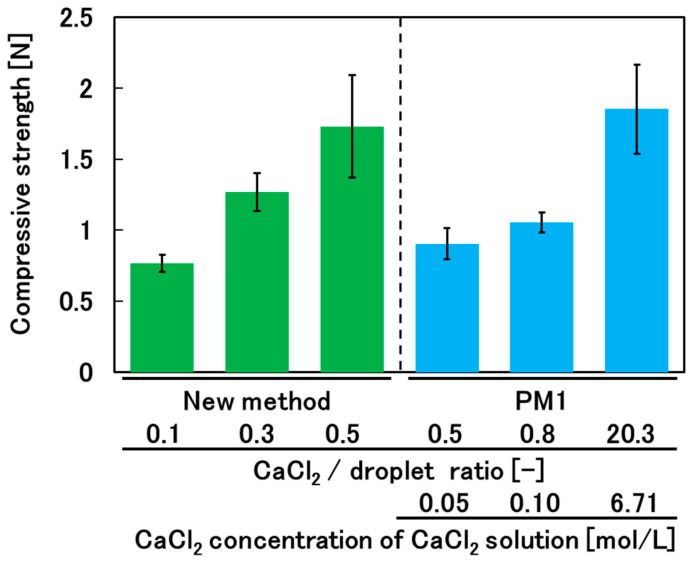
Comparison of the mechanical strengths of calcium alginate hydrogel beads prepared by the new method and by PM1.

We compared the mechanical strengths of calcium alginate hydrogel beads prepared by the new method and by PM1. The strength of a hydrogel bead depends on its diameter. Therefore, it is necessary to prepare hydrogel beads with almost the same diameter. Calcium alginate hydrogel shrinks during the gelation process, and the degree of shrinkage depends on the degree of cross-linking, which depends on the concentration of CaCl_2_. Therefore, we prepared hydrogel beads by varying the volume of the droplet of alginate solution and the weight (and concentration) of CaCl_2_ to produce hydrogel beads with almost the same diameter for all preparation conditions (Table 1). For the new method, the rotation rate was fixed at 200 rpm. Furthermore, based on the results described in Section 3.2, we used a rolling time (gelation time) of over 30 min to ensure sufficient gelation. A comparison of the mechanical strengths of calcium alginate hydrogel beads prepared by the new method and by PM1 is shown in Figure 5. The amount of CaCl_2_ used for PM1 is expressed as the CaCl_2_/droplet ratio to allow comparison with the new method. When the CaCl_2_/droplet ratio was 0.5, the mechanical strength of the hydrogel beads prepared by the new method was higher than that of the hydrogel beads prepared by PM1. This demonstrates that the new method can produce hydrogel beads with greater strength than PM1 while using less CaCl_2_. These results show that the new method is effective for achieving both higher mechanical strength of the hydrogel beads and cost reduction compared with PM1.

### 3.4. Encapsulation Efficiency and Permeability of the Hydrogel Beads

**Figure 6:**
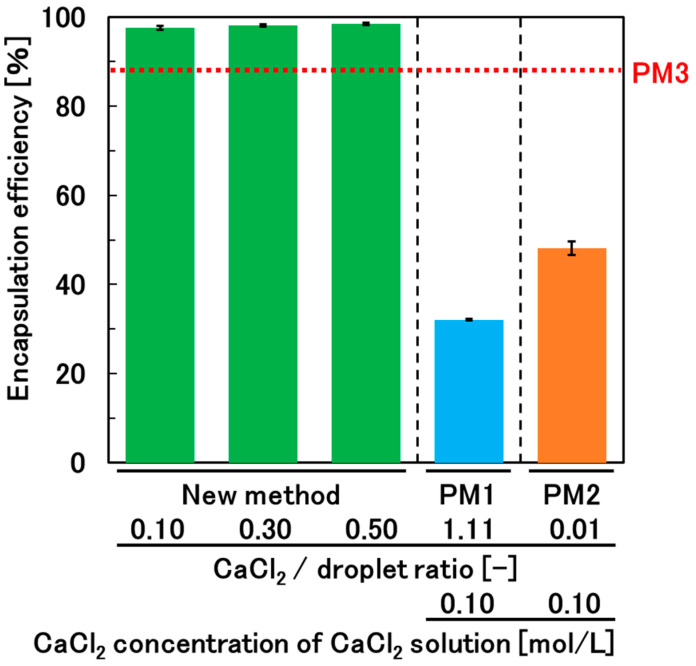
Comparison of the encapsulation efficiency of glucose in the calcium alginate hydrogel beads prepared by the new method and previous methods (PM1–PM3).

We chose glucose (180 Da), a model for low-molecular-weight medicines, as an ingredient to investigate the encapsulation efficiency and permeability of the hydrogel beads. Although the paper reporting PM3 gave the encapsulation efficiency of ingredients, the papers reporting PM1 and PM2 did not specify the encapsulation efficiency of ingredients (e.g., glucose) [15,29]. Therefore, we investigated the encapsulation efficiency of glucose for hydrogel beads prepared not only by the new method, but also by PM1 and PM2. For the new method, the weight of the droplet of glucose-containing alginate solution and rotation rate were fixed at 0.1 g and 200 rpm, respectively. For PM1, after adding a droplet (0.1 g) of glucose-containing alginate solution to 10 mL of CaCl_2_ solution, the gelation time was set to 20 min not only to achieve sufficient gelation but also to minimize glucose leakage (see Figure 4). For the new method and PM2, the gelation time was set to over 30 min to ensure sufficient gelation (see Figure 4). The glucose encapsulation efficiencies for PM1 and PM2 were 32% ± 0% and 48% ± 2%, respectively (Figure 6). The encapsulation efficiency for the new method was 98% ± 0% for all CaCl_2_/droplet ratio conditions, and it was significantly higher than those for PM1 and PM2. The encapsulation efficiency of acetaminophen in PM3 has been reported to be 88% [29]. The slight difference in the molecular weights of glucose (180 Da) and acetaminophen (151 Da) suggests that the new method can more efficiently encapsulate ingredients in hydrogel beads than PM3. This can be attributed to the formation of hydrogel beads with higher mechanical strength by the new method than by PM3, which results from the high degree of cross-linking achieved by directly attaching CaCl_2_ powder, with high molecular density, to the surface of the alginate solution droplets.

**Figure 7:**
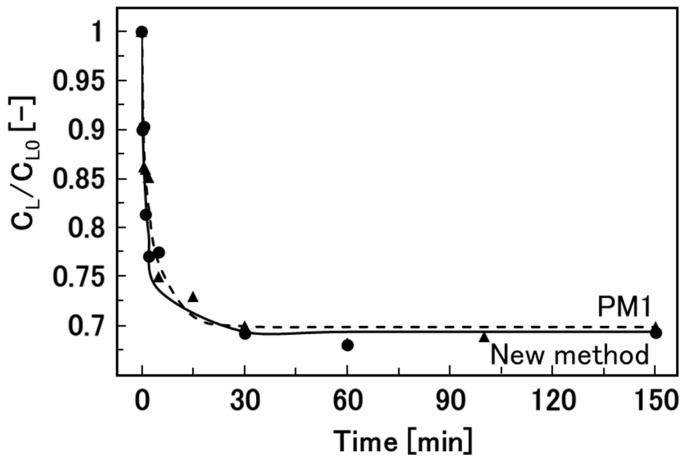
Time courses of the diffusion of glucose from 5 mL of glucose aqueous solution into 2.5 mL of calcium alginate hydrogel beads at room temperature for the new method and PM1. Circles and triangles show our new method and PM1, respectively.

Finally, we measured the effective diffusion coefficients of glucose in hydrogel beads produced by the new method and the gold standard method, PM1 (Figure 7). There was no significant difference in the effective diffusion coefficients for the two methods (Table 2). The values of the effective diffusion coefficients for PM1 were consistent with those in previous reports [31,32]. These results show that the calcium alginate hydrogel beads prepared by the new method are feasible carriers of medicines in DDSs.

## 4. Conclusions

In this study, we demonstrated a new method to produce spherical high-strength calcium alginate hydrogel beads using a smaller amount of CaCl_2_ than conventional methods by rolling an alginate solution droplet on a water-repellent surface with CaCl_2_ powder, a cross-linking agent, and subsequent direct attachment of the CaCl_2_ powder to the droplet. Furthermore, we demonstrated that the encapsulation efficiency of glucose in hydrogel beads prepared by the new method is higher than that in hydrogel beads prepared by previously reported methods. This approach has potential in the ultra-orphan drug field, where it is necessary to encapsulate small amounts of very expensive drugs to treat rare diseases.

## Figures and Tables

**Table 1:** Calcium alginate hydrogel bead preparation conditions for the mechanical strength measurements.

		Volume of the Alginate Droplet [g]	CaCl_2_/Droplet Ratio [-]	CaCl_2_ Concentration [mol/L]	Gel Bead Diameter [mm]
a	New method	0.10	0.1	-	5.0 ± 0.3
b	New method	0.20	0.3	-	5.3 ± 0.1
c	New method	0.30	0.5	-	4.8 ± 0.2
d	PM1	0.08	0.5	0.05	4.8 ± 0.4
e	PM1	0.10	0.8	0.10	5.3 ± 0.2
f	PM1	0.25	20.3	6.71	5.4 ± 0.0

**Table 2:** Effective diffusion coefficients of glucose in calcium alginate hydrogel beads prepared by the new method and by PM1.

	*D*_e_ (m^2^/s)
New method	1.92 × 10^−9^
PM1	1.63 × 10^−9^

## Data Availability

The original contributions presented in this study are included in the article. Further inquiries can be directed to the corresponding author.
