# Correction: Hosokawa et al. Preparation of Alginate Hydrogel Beads on a Superhydrophobic Surface with Calcium Salt Powder to Enhance the Mechanical Strength and Encapsulation Efficiency of Ingredients. *Materials* 2024, *17*, 6027

**DOI:** 10.3390/ma18214916

**Published:** 2025-10-28

**Authors:** Yuhei Hosokawa, Takashi Goshima, Takami Kai, Saki Kobaru, Yoshihiro Ohzuno, Susumu Nii, Shiro Kiyoyama, Masahiro Yoshida, Takayuki Takei

**Affiliations:** 1 Department of Chemical Engineering, Graduate School of Science and Engineering, Kagoshima University, 1-21-40 Korimoto, Kagoshima 890-0065, Japan; k7857533@kadai.jp (Y.H.); tgoshima@cen.kagoshima-u.ac.jp (T.G.); takkai@cb4.so-net.ne.jp (T.K.); inamine@eng.kagoshima-u.ac.jp (S.K.); ohzuno@cen.kagoshima-u.ac.jp (Y.O.); niisus@cen.kagoshima-u.ac.jp (S.N.); myoshida@cen.kagoshima-u.ac.jp (M.Y.); 2 Department of Chemical Science and Engineering, National Institute of Technology, Miyakonojo College, 473-1 Yoshio, Miyazaki 885-8567, Japan; shiroh@miyakonojo-nct.ac.jp

In the original publication [1], the citation referring to reference 12 in the manuscript has been retracted prior to this publication. Concerns were raised on this matter and the following reference was removed from the reference list:

12. Rehman, S.; Madni, A.; Jameel, Q.A.; Usman, F.; Raza, M.R.; Ahmad, F.; Shoukat, H.; Aali, H.; Shafiq, A. Natural polymer-based graphene oxide bio-nanocomposite hydrogel beads: Superstructures with advanced potentials for drug delivery. *AAPS PharmSciTech* **2022**, *23*, 304.

The authors state that the scientific conclusions are unaffected. This correction was approved by the Academic Editor. The original publication has also been updated.
